# A clinical prediction model for post-procedural hemolysis after transcatheter VSD closure in children

**DOI:** 10.3389/fcvm.2026.1864660

**Published:** 2026-09-03

**Authors:** Jingyi Tian, Xinru Gan, Dan Yin, Huichao Sun, Ping Xiang, Mi Li

**Affiliations:** Department of Cardiology, National Clinical Research Center for Child Health and Disorders, Ministry of Education Key Laboratory of Child Development and Disorders, Key Laboratory of Children‘s Vital Organ Development and Diseases of Chongqing Health Commission, National Clinical Key Cardiovascular Specialty, Children’s Hospital of Chongqing Medical University, Chongqing, China

**Keywords:** hemolysis, prediction model, risk factors, transcatheter closure, ventricular septal defect

## Abstract

**Background:**

Transcatheter closure has been widely used for the treatment of ventricular septal defect (VSD) in children. However, post-procedural hemolysis remains a clinically relevant complication, and its underlying mechanisms and risk factors have not been systematically investigated.

**Methods:**

In this single-center retrospective cohort study, children who underwent transcatheter VSD closure at the Children's Hospital of Chongqing Medical University between June 2015 and June 2024 were included. Perioperative clinical data were collected. Univariable and multivariable logistic regression analyses were performed to identify independent risk factors. A prediction model was subsequently developed based on the selected variables and internally validated using bootstrap resampling with optimism correction.

**Results:**

Among 1,427 children who underwent transcatheter VSD closure, hemolysis occurred in 53 cases, yielding an incidence of 3.7%. After exclusions for missing key variables, a cohort of 196 patients (including all 53 hemolysis cases) was used for model development. Multivariable analysis identified occluder diameter, age, residual shunt, and pulmonary valve flow velocity as independent risk factors for post-procedural hemolysis. The model demonstrated good discrimination in the derivation cohort, with an apparent AUC of 0.914 and a bootstrap-corrected AUC of 0.913 (95% CI: 0.865–0.956). Calibration was also satisfactory according to the Hosmer–Lemeshow test. Decision curve analysis demonstrated a net clinical benefit across a threshold probability range of 0%–50%.

**Conclusions:**

Occluder diameter, age, residual shunt, and pulmonary valve flow velocity were identified as independent risk factors for hemolysis after transcatheter VSD closure in children. The developed model showed good discrimination and calibration and may serve as a useful tool for preprocedural risk stratification and postprocedural surveillance. Further external validation and prospective evaluation are warranted.

## 1 Introduction

Ventricular septal defect (VSD) is the most common congenital cardiac anomaly in childhood, accounting for approximately 40% of all congenital heart defects (1). Although surgical repair remains the standard treatment in many regions, percutaneous transcatheter closure has been increasingly adopted in China and other countries because of its minimally invasive nature, shorter hospital stay, and comparable long-term outcomes (2, 3). Despite high procedural success rates, intravascular hemolysis after device closure remains a clinically relevant complication (4). Although most cases are self-limiting, severe or persistent hemolysis can cause acute kidney injury, refractory anemia, and hemodynamic instability, and may necessitate transfusion or surgical device removal. Reported incidence varies widely, from about 0.7% to 15%, depending on study period and occluder type, implying multiple contributing risk factors and incomplete understanding of the underlying mechanisms (5, 6). Hemolysis is generally attributed to high-velocity turbulent flow through residual shunts, where excessive shear stress leads to mechanical erythrocyte destruction (7). However, clinical observations show that not all patients with residual shunts develop hemolysis, while some patients without an obvious residual jet do, suggesting additional determinants. Factors such as device–defect size mismatch, device configuration, and certain anatomical characteristics may alter local hemodynamics and increase shear stress on erythrocytes (8). Prior studies have implicated patient-related, device-related, and procedural risk factors for postprocedural hemolysis—examples include larger defect size, perimembranous aneurysm, pulmonary hypertension, oversized or asymmetric occluders, and residual shunt (9–11). Currently, hemolysis risk assessment relies largely on operator experience, and quantitative predictive tools are lacking. Therefore, this study systematically evaluates perioperative risk factors in children undergoing transcatheter VSD closure with nitinol occluders and integrates anatomical, hemodynamic, and device-related variables to develop and internally validate a nomogram for individualized prediction of postprocedural hemolysis, with the aim of improving preprocedural risk stratification and guiding postoperative management.

## 2 Methods

### 2.1 Study design and ethical approval

This single-center retrospective case-control study reviewed children who underwent transcatheter closure of VSD at the Children's Hospital of Chongqing Medical University. The study protocol was approved by the Ethics Committee of Children's Hospital of Chongqing Medical University (Approval No. 2026-112), and the requirement for informed consent was waived.

### 2.2 Study population and case definition

We screened procedures performed from June 2015 to June 2024. The case group comprised 53 children who developed postoperative intravascular hemolysis, defined as new-onset gross hemoglobinuria (tea- or cola-colored urine) plus at least one laboratory criterion within the prespecified observation window: serum lactate dehydrogenase (LDH) ≥ 2× the upper limit of normal, hemoglobin (Hb) decline >20 g/L from the preprocedural baseline (baseline measured within 24–72 h before the procedure), or new-onset positive occult blood on urinalysis. Two senior cardiovascular physicians independently adjudicated all suspected events to exclude catheter-related trauma, drug-induced hemolysis, or immune-mediated hemolysis. To rigorously address potential selection bias and control for strong confounding factors—specifically the surgical learning curve and temporal variations—a frequency-matched case-control design was employed. The control group included 143 contemporaneous patients who did not develop hemolysis; each control was selected within ±1 month of the corresponding case's procedure date and all procedures were performed by the same interventional team to reduce temporal and operator-related confounding. Comparison of baseline clinical severity between the matched analytical sample (*n* = 196) and the remaining unselected individuals showed no significant systematic bias. The primary outcome window was the early inpatient period (within 72 h after device implantation); late outpatient events after discharge may not have been completely captured.

### 2.3 Inclusion and exclusion criteria

Eligible patients were aged ≤18 years with TTE-confirmed VSD who met indications for catheter closure according to the Chinese expert consensus (12) and who had complete perioperative records. Exclusion criteria were complex congenital heart disease requiring concomitant surgical repair, preexisting hemolytic anemia or autoimmune hematologic disorders, severe hepatic or renal dysfunction, and postoperative Hb decline attributable to other clear causes (e.g., major access-site hematoma, gastrointestinal bleeding, or severe infection).

### 2.4 Procedural protocol and devices

All procedures were performed under general anesthesia following national guidelines and institutional protocol (12–14). Femoral arterial and venous access was obtained and systemic heparinization was administered at 100 U/kg. Preimplantation assessment included left ventricular angiography and transthoracic echocardiography (TTE) to determine VSD location, maximal defect diameter, and relationship to the aortic valve. Devices were released only after satisfactory positioning and confirmation of no adverse interaction with adjacent structures. Immediate postdeployment evaluation included repeat angiography and TTE to assess device position, residual shunt, and valvular regurgitation.

All occluders were domestic modified double-disc nitinol devices (Shanghai Shape Memory Alloy Co., Ltd.; Beijing Starway Medical Co., Ltd.), categorized as symmetric or eccentric by left-disc geometry. Right discs exceeded the waist diameter by 4 mm. Eccentric devices had a left disc that did not extend toward the aortic side but extended 6 mm on the contralateral side; waist length for both types was 3–4 mm.

### 2.5 Data collection and variable definitions

Perioperative data were abstracted from the electronic medical record and PACS and organized into baseline, preoperative, intraoperative, and early postoperative categories. Baseline data included demographics and body surface area (BSA calculated by the Stevenson formula) for indexing diameters (15). Preoperative variables comprised VSD subtype (perimembranous vs. muscular), maximal defect diameter (TTE and angiography; angiographic measurement prioritized for device sizing), defect morphology (membranous aneurysm, multiple defects), hemodynamic parameters (left-to-right shunt velocity, transseptal pressure gradient), associated lesions, and baseline laboratory tests. Intraoperative variables included device type (manufacturer; symmetric vs. eccentric), nominal device diameter, and immediate residual shunt on angiography. Early postoperative monitoring (first 72 h) included TTE measures (presence/width/velocity of residual shunt, gradients) and hemolysis-related tests: Hb and urinalysis were routine for all patients; LDH and bilirubin were obtained selectively when clinically indicated. When multiple measurements were available within the 72 h window, the most clinically relevant abnormal value was used (minimum Hb, peak LDH). We acknowledge that variable monitoring frequency across patients may introduce detection bias.

### 2.6 Statistical analysis and model development

Descriptive statistics were performed in SPSS 25.0; modeling and advanced analyses used R (version 4.5.1). Two-sided *P* < 0.05 indicated statistical significance. Continuous variables were tested for normality by Shapiro–Wilk and compared by independent *t*-test or Mann–Whitney *U*-test as appropriate; categorical variables were compared by chi-square or Fisher's exact test. Candidate predictors (clinical, anatomic, device-related) were first screened by univariable logistic regression. Clinically plausible and literature-supported variables entered a multivariable logistic regression; multicollinearity was assessed by variance inflation factor (VIF < 5 considered acceptable). Backward stepwise selection by AIC yielded a parsimonious multivariable model; LASSO with 10-fold cross-validation was performed in parallel to confirm variable selection. Events-per-variable considerations were applied given 53 events.

### 2.7 Internal validation and performance metrics

Bootstrap resampling (with replacement) for 1,000 iterations provided optimism-corrected estimates. Model performance was evaluated by discrimination (ROC AUC with 95% CI), calibration (calibration plot and Hosmer-Lemeshow test), overall metrics (bootstrap-corrected accuracy, sensitivity, specificity), and clinical utility assessed via decision curve analysis and clinical impact curves across threshold probabilities.

## 3 Results

### 3.1 Baseline clinical characteristics

Between June 2015 and June 2024, 1,427 patients underwent percutaneous closure of VSD at the participating center. Postoperative hemolysis occurred in 53 cases, corresponding to an incidence of 3.7%. The final analytic cohort comprised 196 pediatric patients, including 53 in the hemolysis group and 143 in the non-hemolysis group. For the entire cohort, the median age was 43.0 (IQR 30.8–65.0) months; 94 patients (48.0%) were male and 102 (52.0%) were female. Detailed baseline comparisons are shown in Table 1.

**Table 1:** Comparison of baseline clinical characteristics between the hemolysis and non-hemolysis groups.

Parameter	Total (*n* = 196)	Non-hemolysis group (*n* = 143)	Hemolysis group (*n* = 53)	*P*-value
Demographics				
Gender (Male), *n* (%)	94 (47.96%)	64 (44.76%)	30 (56.60%)	0.153
Age (months)	43.00 (30.75,65.00)	45.00 (36.00,68.00)	36.00 (28.00,61.00)	0.011
VSD Characteristics				
VSD Subtype, *n* (%)				0.780
Perimembranous	172 (87.76%)	126 (88.11%)	46 (86.79%)	
Intracristal	24 (12.24%)	17 (11.89%)	7 (13.21%)	
Multiple defects (Yes), *n* (%)	84 (42.86%)	62 (43.36%)	22 (41.51%)	0.249
Membranous aneurysm (Yes), *n* (%)	63 (32.14%)	42 (29.37%)	21 (39.62%)	0.151
Aortic valve prolapse (Yes), *n* (%)	135 (68.88%)	98 (68.53%)	37 (69.81%)	0.857
VSD diameter/BSA (mm/m^2^)	9.47 (6.77,12.19)	8.52 (6.39,11.42)	11.71 (9.99,13.32)	<0.001
Left-to-right shunt velocity (m/s)	4.08 (3.88,4.25)	4.08 (3.92,4.24)	4.01 (3.72,4.30)	0.395
Left-to-right shunt pressure gradient (mmHg)	66.47 (61.00,73.12)	67.00 (62.00,71.60)	65.00 (56.00,75.00)	0.545
Occluder Characteristics				
Occluder Shape, *n* (%)				0.065
Symmetric	36 (18.37%)	25 (17.48%)	11 (20.75%)	
Eccentric	160 (81.63%)	118 (82.52%)	42 (79.25%)	
Manufacturer, *n* (%)				0.092
Starway Medical (Beijing)	124 (63.27%)	84 (58.74%)	40 (75.47%)	
Shape Memory (Shanghai)	72 (36.73%)	59 (41.26%)	13 (24.53%)	
Occluder diameter (mm)	7.00 (6.00,9.00)	7.00 (6.00,8.00)	10.00 (8.00,12.00)	<0.001
Echocardiographic Data				
Tricuspid regurgitation (Moderate or more), *n* (%)	14 (7.14%)	5 (3.50%)	9 (16.98%)	0.008
Mitral regurgitation (Mild), *n* (%)	171 (87.24%)	121 (84.62%)	50 (94.34%)	0.075
Pulmonary regurgitation (Mild), *n* (%)	151 (77.04%)	108 (75.52%)	43 (81.13%)	0.586
Aortic regurgitation (Mild), *n* (%)	78 (39.80%)	55 (38.46%)	23 (43.40%)	0.370
Pulmonary valve flow velocity (m/s)	1.13 (1.03,1.32)	1.10 (1.02,1.21)	1.36 (1.15,1.47)	<0.001
Aortic valve flow velocity (m/s)	1.08 (0.99,1.17)	1.06 (0.98,1.15)	1.13 (0.99,1.23)	0.128
Tricuspid valve flow velocity (m/s)	0.75 (0.67,0.84)	0.75 (0.67,0.83)	0.74 (0.68,0.87)	0.559
Mitral valve flow velocity (m/s)	1.06 (0.97,1.19)	1.04 (0.95,1.14)	1.14 (1.05,1.28)	<0.001
Procedural Outcomes				
Residual shunt (Yes), *n* (%)	46 (23.47%)	20 (13.99%)	26 (49.06%)	<0.001
RVSP (mmHg)	24.00 (22.00,27.00)	24.00 (22.00,27.00)	26.00 (23.00,30.00)	<0.001
Preoperative Hematology				
RBC (×1,012/L)	4.67 ± 0.39	4.69 ± 0.40	4.61 ± 0.35	0.407
Hemoglobin (g/L)	125.00 (120.00,131.00)	126.00 (120.50,131.00)	125.00 (120.00,128.00)	0.175

Categorical variables are expressed as *n* (%); continuous variables with normal distribution are expressed as Mean ± SD, and non-normally distributed continuous variables are expressed as Median (Interquartile Range).

VSD, ventricular septal defect; BSA, body surface area; RVSP, right ventricular systolic pressure; RBC, red blood cell count; SD, standard deviation.

Median age was lower in the hemolysis group than in the non-hemolysis group (36.0 vs. 45.0 months, *P* = 0.011). With respect to defect and device measurements, the hemolysis group had a larger normalized VSD diameter (median 11.71 vs. 8.52 mm/m2, *P* < 0.001) and a larger occluder diameter (median 10.00 vs. 7.00 mm, *P* < 0.001).

Echocardiography revealed that moderate or greater tricuspid regurgitation was more frequent among patients with hemolysis (16.98% vs. 3.50%, *P* = 0.008). Both pulmonary valve flow velocity and mitral valve flow velocity were higher in the hemolysis group (PV: median 1.36 vs. 1.10 m/s, *P* < 0.001; MV: median 1.14 vs. 1.04 m/s, *P* < 0.001).

Procedural outcomes showed a markedly higher incidence of residual shunt in the hemolysis group compared with the non-hemolysis group (49.06% vs. 13.99%, *P* < 0.001). The hemolysis group also had a higher right ventricular systolic pressure (RVSP) (median 26.00 vs. 24.00 mmHg, *P* < 0.001). No significant between-group differences were observed for sex distribution, VSD subtype, shunt velocity, occluder shape, or preoperative hematologic indices (all *P* > 0.05).

### 3.2 Risk factor analysis

Univariate logistic regression identified several variables associated with postoperative hemolysis (Table 2). The largest univariate odds ratios were observed for mitral valve flow velocity (OR = 44.79, 95% CI 7.19–278.82, *P* < 0.001), pulmonary valve flow velocity (OR = 20.45, 95% CI 5.30–78.93, *P* < 0.001), and presence of a residual shunt (OR = 5.92, 95% CI 2.89–12.13, *P* < 0.001). Additional univariately significant factors included younger age, larger occluder and VSD diameters, higher tricuspid regurgitation grade, elevated RVSP, and occluder manufacturer (all *P* < 0.05). Gender, VSD subtype, and preoperative hematologic parameters were not significant (all *P* > 0.05).

**Table 2:** Univariate and multivariate logistic regression analysis of risk factors for postoperative hemolysis.

Parameter	Univariate Analysis		Multivariate Analysis	
	OR (95% CI)	*P*-value	OR (95% CI)	*P*-value
Gender (Female vs. Male)	0.62 (0.33–1.17)	0.142	—	—
Age	0.98 (0.97–1.00)	0.018	0.96 (0.93–0.98)	0.001
Occluder manufacturer	0.46 (0.23–0.94)	0.033	—	—
Occluder diameter	1.85 (1.52–2.24)	<0.001	2.19 (1.62–2.97)	<0.001
VSD diameter/BSA	1.24 (1.13–1.36)	<0.001	0.89 (0.76–1.04)	0.153
Tricuspid regurgitation	5.65 (1.80–17.73)	0.003	4.27 (0.75–24.23)	0.101
Mitral regurgitation	3.03 (0.87–10.58)	0.082	—	—
Pulmonary valve flow velocity	20.45 (5.30–78.93)	<0.001	4.89 (1.23–19.43)	0.024
Mitral valve flow velocity	44.79 (7.19–278.82)	<0.001	—	—
Residual shunt	5.92 (2.89–12.13)	<0.001	3.16 (1.23–8.12)	0.017
RVSP	1.10 (1.03–1.18)	0.004	—	—

Parameters: Age is measured in months; Occluder and VSD diameters are in mm; Flow velocity is in m/s; RVSP is in mmHg.

Symbols: “—” indicates that the variable was not included in the final multivariate Logistic regression model.

Statistical significance: *P*-value <0.05 was considered statistically significant.

For multivariate analysis, backward stepwise selection using the Akaike Information Criterion (AIC) initially produced a six-variable model containing occluder diameter, age, residual shunt, pulmonary valve flow velocity, tricuspid regurgitation, and VSD diameter/BSA. Multicollinearity diagnostics yielded variance inflation factors between 1.00 and 1.82, indicating no concerning collinearity. In the preliminary model, tricuspid regurgitation (*P* = 0.101) and VSD diameter/BSA (*P* = 0.153) were not statistically significant; exclusion of these two variables improved model parsimony without a significant loss of fit (likelihood ratio test: *χ*^2^ = 2.15, *P* = 0.341). Therefore, age, occluder diameter, residual shunt, and pulmonary valve flow velocity were retained as the core independent risk factors. Consequently, with 53 confirmed hemolytic events and 4 final predictors, the event-per-variable (EPV) ratio of our final multivariable model is 13.25 (53/4). This safely exceeds the standard methodological recommendation of EPV≥10, ensuring the numerical stability of the regression coefficients and minimizing the risk of over-fitting.

In the final multivariate model (Table 2), age was protective (OR = 0.96, 95% CI 0.93–0.98, *P* = 0.001), equating to an approximate 4% reduction in odds of hemolysis per additional month of age. Occluder diameter was an independent risk factor (OR = 2.19, 95% CI 1.62–2.97, *P* < 0.001); each 1 mm increase in occluder diameter was associated with an approximate 2.19-fold increase in odds. Presence of a residual shunt was associated with higher risk (OR = 3.16, 95% CI 1.23–8.12, *P* = 0.017). Pulmonary valve flow velocity remained a significant predictor (OR = 4.89, 95% CI 1.23–19.43, *P* = 0.024), with odds increasing appreciably per additional 1 m/s.

**Figure 1:**
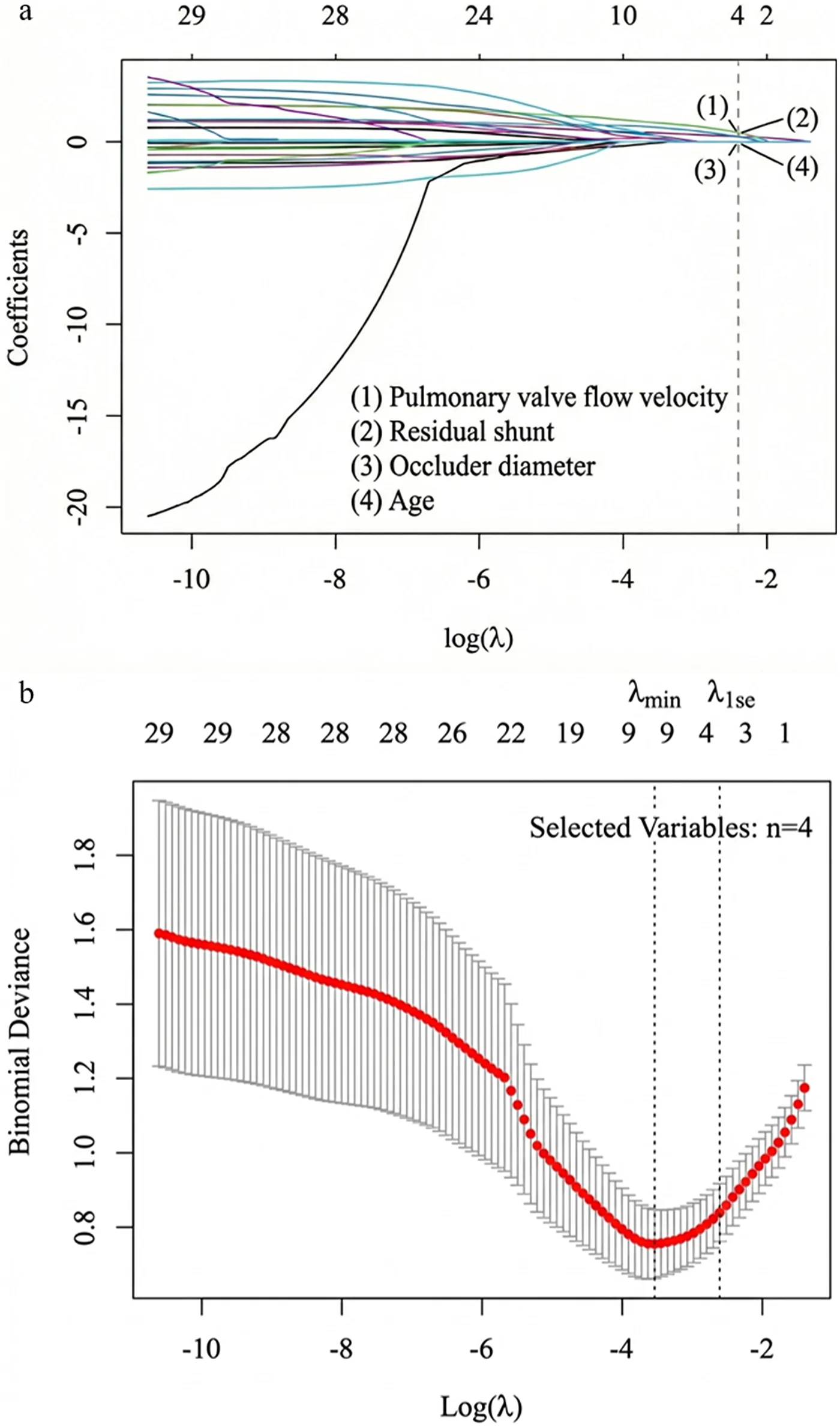
Variable selection and model construction using the LASSO regression analysis. **(a)** LASSO coefficient path diagram. The plot shows the relationship between the coefficients of potential risk factors and the penalty parameter [log(*λ*)]. Each colored line represents a candidate variable. As the penalty increases [log(*λ*) moves to the right], non-significant coefficients are shrunk to zero. The vertical dashed line indicates the optimal lambda value selected, resulting in four key risk factors: (1) Pulmonary valve flow velocity, (2) Residual shunt, (3) Occluder diameter, and (4) Age. **(b)** Tuning parameter (*λ*) selection via 10-fold cross-validation. The binomial deviance is plotted against log(*λ*). The red dots represent the mean binomial deviance for each value of *λ*, with error bars indicating the standard deviation (SD). The two vertical dashed lines represent the minimum deviance (*λ*min) and the one standard error criterion (*λ*1se). In this study, *λ*1se was used to identify the most parsimonious model with four risk factors (*n* = 4), ensuring both predictive accuracy and model robustness. LASSO, least absolute shrinkage and selection operator; SE, standard error; *λ*, lambda (the regularization parameter).

To evaluate the stability of variable selection, LASSO regression with 10-fold cross-validation determined the optimal penalty parameter *λ* (Figure 1a). The coefficient path (Figure 1b) indicated that, as *λ* increased, only the coefficients for the four retained risk factors remained non-zero while others were driven to zero. This result is consistent with the AIC-based selection and supports the robustness of the chosen risk factors.

### 3.3 Static and dynamic nomograms

**Figure 2:**
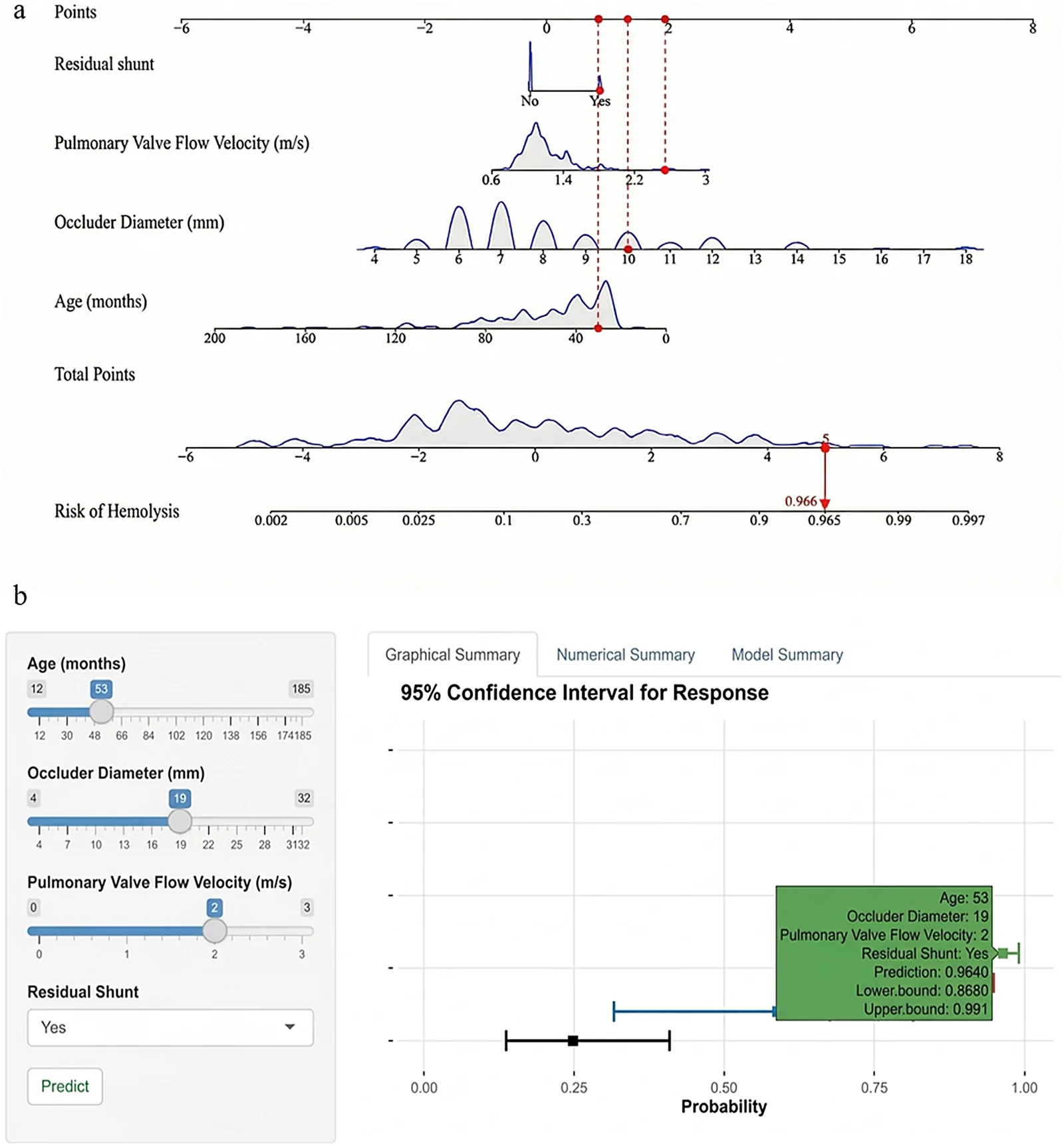
Clinical visualization tools for predicting the risk of postoperative hemolysis. **(a)** Static Nomogram. This tool allows for the individualized assessment of hemolysis risk by summing the points assigned to each of the four independent risk factors: residual shunt, pulmonary valve flow velocity (m/s), occluder diameter (mm), and age (months). To use the nomogram, a vertical line is drawn from each risk factor's value to the “Points” axis at the top. The sum of these points is then located on the “Total Points” axis, and a vertical line is projected downward to the “Risk of Hemolysis” scale to determine the estimated probability. The red dots and arrows illustrate an example case with a predicted risk of 0.966. **(b)** Dynamic Nomogram interface. An interactive, web-based version of the prediction model was developed to facilitate real-time clinical decision-making. The interface features input sliders and dropdown menus for patient risk factors (left panel). Upon clicking the “Predict” button, the tool automatically calculates the predicted probability of hemolysis and displays it with a 95% confidence interval (95%CI) in both graphical and numerical formats (right panel). This dynamic tool enhances the precision and efficiency of risk evaluation in a clinical setting.

Using the four independent risk factors (age, occluder diameter, residual shunt, and pulmonary valve flow velocity), we constructed a static nomogram for individualized prediction of postoperative hemolysis risk (Figure 2a). Each predictor maps to a point score on the top axis; the summed total score projects to a predicted probability of hemolysis. Density curves on the nomogram depict the distribution of each predictor within the study cohort.

To facilitate real-time decision making, we developed a web-based dynamic nomogram (Figure 2b). Clinicians can generate individualized risk predictions with accompanying 95% confidence intervals by adjusting sliders for continuous inputs—age (months), occluder diameter (mm), and pulmonary valve flow velocity (m/s)—or by selecting categorical options (residual shunt). In a high-risk example (age 53 months; occluder 19 mm; pulmonary valve flow 2 m/s; residual shunt present), the model yields a predicted probability of hemolysis of 0.964 (95% CI 0.868–0.991). The dynamic interface therefore provides point estimates with quantified uncertainty to support individualized postoperative management.

### 3.4 Performance validation and clinical utility

**Figure 3:**
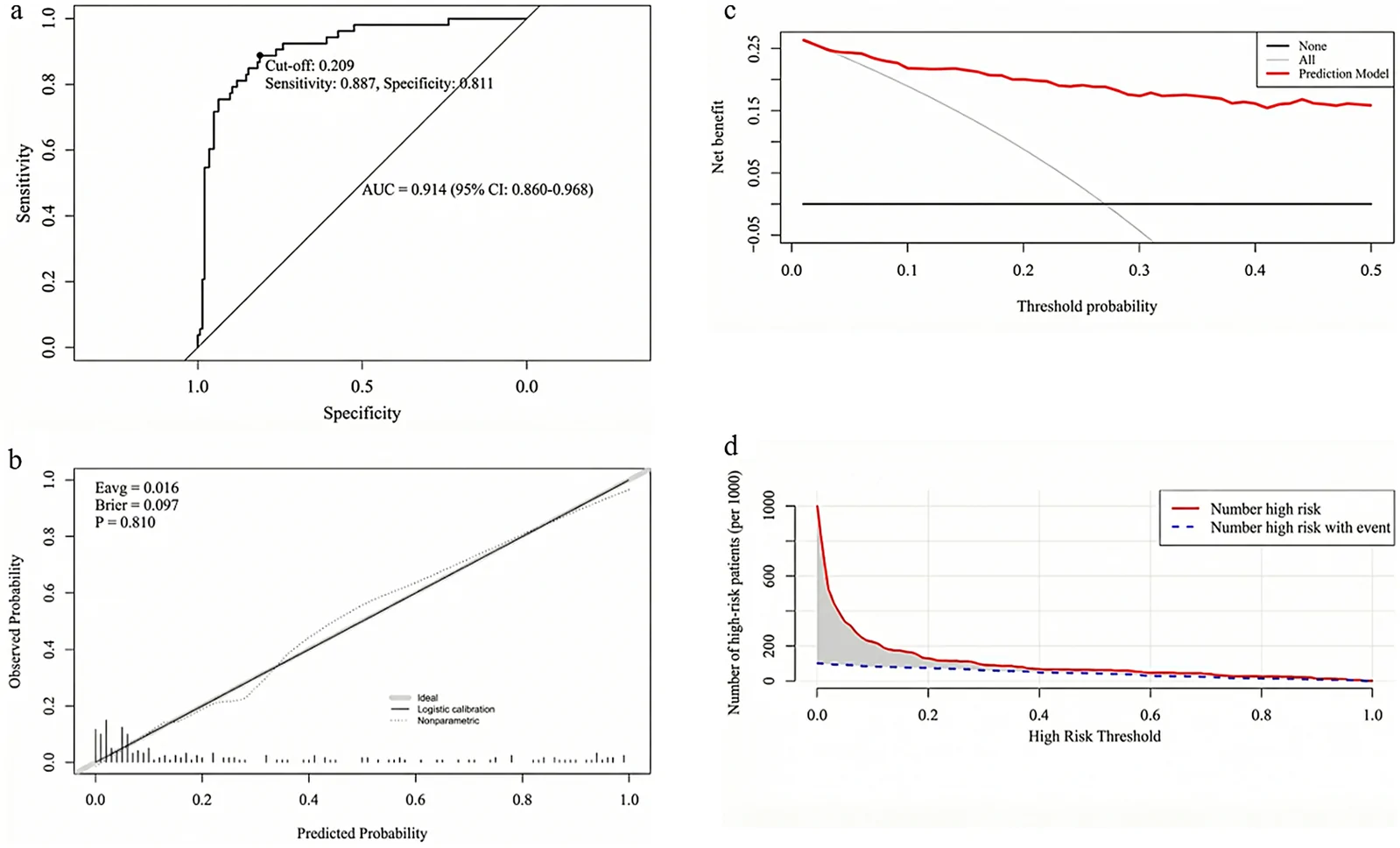
Multi-dimensional evaluation of the prediction model's performance and clinical utility. **(a)** Receiver operating characteristic (ROC) curve. The ROC curve evaluates the discriminative ability of the risk prediction model. The area under the curve (AUC) is 0.914 (95%CI:0.860–0.968), indicating excellent discrimination. At the optimal risk threshold (0.209), the model achieves a sensitivity of 0.887 and a specificity of 0.811. **(b)** Calibration plot. This plot demonstrates the agreement between predicted probability and observed outcomes. The diagonal grey line represents ideal prediction. The model shows excellent calibration with low average error (Eavg​=0.016), a Brier score of 0.097, and a non-significant *P* value (*P* = 0.810), indicating good fit. **(c)** Decision curve analysis (DCA). DCA evaluates the clinical net benefit of the prediction model across various threshold probabilities. The model (solid red line) provides a consistently higher net benefit than either treating all (descending grey line) or treating none (horizontal black line) across a wide range of practical thresholds (from nearly 0 to 0.5). This threshold range is highly relevant as it spans the lower risk boundaries where clinicians are most likely to initiate prophylactic interventions for post-VSD closure hemolysis, confirming its high clinical relevance. **(d)** Clinical impact curve (CIC). The CIC visualizes the model's clinical applicability by estimating patient stratification per 1,000 cases. The solid red line indicates the number of patients classified as high risk, while the dashed blue line represents high-risk patients who actually experience the event. The close proximity of these curves at most thresholds indicates effective identification of true positives while minimizing false alarms. ROC, receiver operating characteristic; AUC, area under the curve; CI, confidence interval; DCA, decision curve analysis; CIC, clinical impact curve.

Model performance was assessed through discrimination, calibration, and clinical utility analyses (Figure 3). ROC analysis showed excellent discrimination with area under the curve (AUC) = 0.914 (95% CI 0.860–0.968, Figure 3a). Calibration was strong (Figure 3b): mean absolute error (Eavg) = 0.016, Brier score = 0.097, and Hosmer-Lemeshow test non-significant (*P* = 0.810). Internal validation with 1,000 bootstrap resamples produced a bias-corrected AUC of 0.913 (95% CI 0.865–0.956) with sensitivity 0.808 and specificity 0.872 (Table 3).

**Table 3:** Internal validation of the hemolysis prediction model using the bootstrap method (*n* = 1,000 resamples).

Performance Metric	Apparent Performance	Bias-corrected Performance (Mean)	95% Confidence Interval
AUC	0.914	0.913	0.865–0.956
Accuracy	0.832	0.830	0.791–0.923
Sensitivity	0.887	0.852	0.712–0.962
Specificity	0.811	0.808	0.754–0.966
PPV	0.635	0.631	0.553–0.897
NPV	0.951	0.942	0.890–0.984

The Bootstrap method with 1,000 resamples was employed to evaluate the internal validity and stability of the predictive model.

AUC, area under the receiver operating characteristic curve; PPV, positive predictive value; NPV, negative predictive value.

Decision curve analysis indicated that the model provides net benefit across a wide range of threshold probabilities (0–0.5) relative to “treat-all” or “treat-none” strategies (Figure 3c). In clinical practice, given that post-procedural hemolysis is an acute and severe complication, clinicians typically tolerate a lower risk threshold (e.g., 5%–30%) to trigger aggressive surveillance. Therefore, the 0%–50% net benefit range perfectly encapsulates the realistic clinical decision-making window, demonstrating strong practical utility. The clinical impact curve demonstrated that the number of predicted high-risk patients closely tracked observed hemolysis events (Figure 3d), supporting the model's potential utility for identifying patients who may benefit from targeted prevention or intensified surveillance.

## 4 Discussion

In this single-center retrospective study, we systematically analyzed the occurrence of postprocedural intravascular hemolysis following transcatheter closure of VSD in children and developed an individualized risk model that included four independent predictors: younger age, larger occluder diameter, higher pulmonary valve flow velocity (PV), and presence of residual shunt. The incidence of hemolysis in our cohort was 3.7%, which lies within previously reported ranges of 0.7%–15% (16) and is lower than the 4.7%–7.1% reported in a national guideline update (12). This reduced incidence may reflect increasing procedural experience and more detailed preprocedural anatomic assessment. As a single-center analysis conducted under standardized operative protocols, the study also benefits from lower heterogeneity in perioperative management than typically encountered in multicenter series.

Mechanistically, the four predictors converge on a single pathophysiologic pathway: local hemodynamic forces determine whether red blood cells (RBCs) undergo mechanical injury. PV was the strongest independent predictor [adjusted OR (aOR) = 4.89], suggesting that elevated transvalvular velocities create a vulnerable hemodynamic milieu. From a fluid-mechanics perspective, hemolysis reflects both instantaneous shear thresholds and cumulative exposure to sublethal shear. The shear stress required for immediate RBC rupture in steady laminar flow is on the order of 400 Pa (17, 18), but sublethal membrane injury occurs at much lower shear levels; sustained shear of approximately 30–50 Pa is sufficient to produce cumulative fatigue and loss of deformability (19). Patients with elevated baseline PV may therefore have RBCs already stressed within this sublethal window. After occluder implantation, even minor local reductions in cross-sectional area can increase velocity by continuity and push local shear into the lethal range. Because kinetic energy scales with the square of velocity, an increase of PV from 2 m/s to 3 m/s raises flow kinetic energy by a factor of 2.25, amplifying both impact forces and turbulence intensity (20). Biologically, platelets respond to shear at lower thresholds than RBCs; acute platelet consumption may thus function as an early sentinel of pathologically elevated local shear (21).

This hemodynamic framework also explains why apparently “trace” residual shunts can be clinically harmful. According to Bernoulli principles, a substantial pressure gradient across a small orifice converts pressure energy into kinetic energy, producing high-velocity jets; consequently, a small shunt volume can still generate damaging peak velocities. Clinical evidence indicates that jet peak velocity and focal impact, rather than bulk shunt volume, better predict refractory hemolysis (22). High-velocity jets entering relatively quiescent chambers disintegrate into a hierarchy of vortices: per Kolmogorov turbulence theory, energy cascades from large eddies to smaller scales and ultimately to the Kolmogorov microscale (approximately 5–10 μm, comparable to RBC diameter), where intense viscous dissipation produces multi-directional, high-frequency shear fields capable of mechanically fragmenting RBCs (23). In anatomies with eccentric or irregular defects (for example, perimembranous VSD or membranous aneurysm), crescentic gaps adjacent to occluder edges can direct narrow jets toward cardiac structures or device components, superimposing collision trauma on shear stress. High-velocity impingement also washes away platelets and fibrin, impeding endothelialization and fostering a “high-flow, impaired-repair, persistent-hemolysis” cycle (24–26). These considerations emphasize that assessment should include peak jet velocity and jet directionality in addition to residual volume.

Occluder selection is the primary modifiable factor under the operator's control. In our model, each 1 mm increase in occluder diameter was associated with an approximate 119% increase in hemolysis risk (aOR = 2.19). This warns against assuming that larger occluders are uniformly advantageous; oversizing to achieve immediate complete closure expands the device's exposed surface and alters local flow geometry, increasing the frequency of RBC–occluder collisions (27). The occluder mesh functions as a turbulence generator: no-slip boundary conditions create a boundary layer and a sharp velocity gradient between the stagnant fluid adjacent to the metal and the faster flow through mesh pores, producing intense local shear. As RBCs traverse these gradients, differential velocities across the cell produce stretch and shear stresses that destabilize membrane mechanics (8, 28–30). Larger occluders enlarge and complicate disturbed-flow regions, extending RBC residence time in sublethal stress ranges and accelerating cumulative fatigue toward rupture. Accordingly, we advocate an anatomically precise sizing strategy rather than aggressive oversizing—typically selecting occluders only 1–2 mm larger than the defect when anatomically appropriate—and prioritizing geometric fit over an immediate angiographic “perfect” closure (13).

Translating these insights into practice, our model shifts risk assessment from a single focus on residual shunt to an integrated approach spanning preprocedural decision-making, intraoperative occluder selection, and postoperative surveillance (31, 32). For patients with model-predicted high risk (for example, predicted probability > 50%), we recommend multidisciplinary preoperative review, explicit family counseling about hemolysis risk and surgical alternatives, and heightened intraoperative vigilance. For markedly elevated PV (e.g., >3 m/s), clinicians should recognize the predisposed “stress-fatigued” RBC state, favor conservative occluder sizing, immediately reassess residual jet peak velocity and direction after deployment, and maintain a low threshold to consider occluder repositioning or replacement when a narrow, high-velocity, eccentric jet is present (33). Intraoperative and early postoperative monitoring for high-risk patients should include frequent urinalysis (for example, every 6–8 h), serial hemoglobin and LDH measurements, and platelet counts as a potential sentinel marker. Postdischarge follow-up at predefined intervals (for example, 1 week and 1 month) with echocardiographic reassessment of PV and residual jets is advisable.

### 4.1 Study limitations

As a retrospective single-center study, residual confounding and detection bias are possible: cases frequently underwent more intensive testing after the event, which can create asymmetry in capturing transient or subclinical hemolysis. We attempted to mitigate this by using the most abnormal value within a standardized 72 h window and by independent adjudication, but unmeasured operator- or perioperative-care differences may remain. Statistical precision is limited by 53 events; although PV showed a strong point estimate (aOR = 4.89), the confidence interval was wide (95% CI 1.23–19.43), indicating imprecision and the need for validation in larger cohorts with robust resampling or small-sample bias correction. Late or delayed hemolysis beyond the early inpatient period may be underdetected; prospective studies with extended follow-up are required. Finally, while we link clinical observations to fluid-mechanics hypotheses, direct quantification of local shear by advanced imaging or patient-specific CFD was not available; future studies combining 4D flow MRI or CFD with clinical data would strengthen causal inference.

## 5 Conclusions

In summary, PV, occluder diameter, residual shunt, and younger age emerged as key determinants of post-closure hemolysis. Integrating preprocedural hemodynamic assessment, anatomically precise occluder selection, and targeted monitoring may reduce hemolysis risk. Prospective validation and mechanistic imaging studies are needed to refine thresholds and translate the model into clinical decision support.

## Data Availability

The original contributions presented in the study are included in the article/Supplementary Material, further inquiries can be directed to the corresponding author.
